# High-volume hospitals do not perform better than low-volume hospitals in septic and aseptic revision total hip arthroplasty: an analysis of re-revision risk and mortality based on the Dutch Arthroplasty Register

**DOI:** 10.2340/17453674.2025.44331

**Published:** 2025-08-15

**Authors:** Marije C VINK, Rinne M PETERS, Bart VAN DOOREN, Amarens DEEN, Liza N VAN STEENBERGEN, B Wim SCHREURS, Wierd P ZIJLSTRA

**Affiliations:** 1 Department of Orthopedic Surgery, Medical Center Leeuwarden, the Netherlands; 2 Department of Orthopedic Surgery, University Medical Center Groningen, the Netherlands; 3 Department of Orthopedic Surgery, Flinders Medical center, Adelaide, Australia; 4 Dutch Arthroplasty Register (LROI), ‘s Hertogenbosch, the Netherlands; 5 Department of Orthopedic Surgery, Radboud UMC, Nijmegen, The Netherlands

## Abstract

**Background and purpose:**

Revision total hip arthroplasty (rTHA) is a complex procedure that may benefit from centralization. We examined the association between annual hospital volume of rTHA and re-revision risk and mortality.

**Methods:**

We included all rTHAs between 2007 and 2022 in general hospitals, registered in the Dutch Arthroplasty Register (LROI; n = 12,515). Hospitals were categorized into low (< 25 rTHA/year) or high volume (≥ 25 rTHA/year). Competing-risk analyses and Cox proportional hazard regression analyses were performed to assess implant re-revision and Kaplan–Meier survival analysis for mortality. Results were stratified into septic (permanent Girdlestone, 1-stage, and 2-stage revisions) and aseptic first revisions.

**Results:**

1-stage septic revisions showed a higher risk of re-revision in high-volume hospitals (hazard ratio [HR] 1.6, 95% confidence interval [CI] 1.1–2.4). We found no difference in re-revision risk after DAIR (HR 1.1, CI 0.9–1.3). 2-stage septic revisions were more often performed in high-volume hospitals (5% vs 2%). There was no statistical difference in re-revision rates between hospitals after revision for aseptic loosening (HR 1.1, CI 0.9–1.4), dislocation (HR 1.1, CI 0.9–1.4), and periprosthetic fractures (HR 1.1, CI 0.8–1.5). Mortality showed no differences between groups, neither for septic nor aseptic revisions.

**Conclusion:**

There was no difference between high-volume hospitals and low-volume hospitals regarding risk for re-revision after aseptic loosening, dislocation and periprosthetic fracture, and septic DAIR and mortality. In high-volume hospitals, 1-stage septic revisions was associated with a significantly higher re-revision risk. 2-stage revisions are more frequent in high-volume hospitals, indicating more complex pathology.

As the incidence of total hip arthroplasties (THA) rises and the life expectancy of patients improves, there is growing concern regarding the increasing incidence of revision surgery of failed THA [1,2]. In the Netherlands, nowadays over 3,500 hip revision procedures are performed annually and, based on current trends, it is anticipated this number will continue to grow [3]. In addition, the patient population undergoing a primary THA is becoming increasingly heterogeneous, with a rising number of young and obese patients, who have a higher likelihood of encountering revisions over time [4,5].

Some of the revision THA (rTHA) procedures are known to be more technically challenging than primary THA, with higher complication and mortality rates [6-9]. Ong et al. (2010) reported a risk of revision of 4% within 5 years in primary THA, compared with 19% re-revisions in rTHA [10]. The Dutch Arthroplasty Register reports a comparable crude cumulative risk of re-revision of 14% after 5 years [3].

With the rising incidence of rTHA, there is a growing discussion on centralization and specialization. Previous studies have shown that hospitals performing a higher number of orthopedic procedures, in both primary and revision arthroplasty, tend to have better outcomes, including lower complication rates, reduced mortality, and improved patient satisfaction [11-16]. However, Van Rensch et al. (2023) reported no association between hospital volume and re-revision rate in septic and aseptic total knee revision arthroplasty in the Netherlands [17]. In the Netherlands, the relation between hospital volume rTHA and re-revision rate remains unknown.

We therefore investigated the association between annual rTHA hospital volume and the risk of re-revision, as well as mortality. We differentiated between septic and aseptic first revisions and revision due to dislocation and periprosthetic fracture.

## Methods

### Study design and data sources

This is a register study based on data from the Dutch Arthroplasty Register (LROI), which is a nationwide population-based register, with high validity and completeness (98–99% for primary THA, 97–98% for revision THA) [3,18], containing data of primary arthroplasties and revisions in the Netherlands since 2007. Completeness of PJIs in the LROI is 33%, including (suspicion of) PJIs with and without revision [19]. Patient and procedure characteristics were recorded. Data from the LROI was matched with the national insurance database on healthcare [20], in order to obtain information on the vital status and date of death of registered patients. The Dutch healthcare system is based on mandatory health insurance for all residents, providing access to essential medical services. Patients may choose freely between public and private hospitals. According to the definition used by the LROI, public hospitals are healthcare institutions that are owned, managed, and financed by the government or a public authority. In contrast, private hospitals are independent healthcare providers that often specialize in a specific type of treatment, medical discipline, or patient group. Orthopedic procedures are performed in both public and private hospitals in the Netherlands. Patient populations differ, with private hospitals more commonly treating younger, healthier individuals, while public hospitals care for patients with more comorbidities [21].

### Inclusion and exclusion criteria

We included all first rTHAs performed between 2007 and 2022 and followed them until the first re-revision, death, or end of follow-up. Patients younger than 18 years were excluded. To create more representative and generalizable results, only general hospitals were analyzed in this study. University and private hospitals were excluded. Patients treated in hospitals with unknown annual volume were also excluded (e.g., first revision in 2007, the first year of national registration). The results were reported in accordance with the STROBE guidelines [22].

### Data collection

Revision was defined as the exchange, addition, or removal of at least 1 prosthesis component. Re-revision refers to the surgical intervention involving replacement, addition, or removal of at least 1 component subsequent to a prior first revision surgery. Major revision is defined as revision of at least the acetabular or femoral component, while minor revision is defined as the revision of only inlay and/or femoral head component.

Volume groups were defined based on previous studies, categorizing hospitals as high volume or low volume according to the number of revision procedures performed at each hospital in the year prior to the revision procedure. In this study, a hospital was categorized as high volume when 25 or more revision cases were registered in the year prior to that individual procedure, and categorized as a low-volume hospital when less than 25 procedures were registered in that hospital in the preceding year [14]. The procedure was categorized as high or low volume based on the location of the first revision. If a re-revision takes place after referral to a different hospital, the procedure remained linked to the initial hospital. We calculated the revision-to-primary ratio per hospital.

The results were stratified based on septic and aseptic first revisions and additionally subdivided into: 1-stage septic revision (with exchange of cup and/or stem), 2-stage septic revision, permanent septic Girdlestone (removal of all components), and minor septic revision (debridement and implant retention with exchange of mobile parts, DAIR) for septic first rTHA. Aseptic revisions procedures were subdivided into aseptic loosening, periprosthetic fracture, and dislocation.

The primary outcome measure was second revision, expressed as crude re-revision rate and (adjusted) hazard ratio (HR), determined separately for septic and aseptic first revisions. The secondary outcome was mortality.

### Statistics

A competing risk analysis was performed, to assess re-revision rate. Cumulative incidence of re-revision of both volume groups was determined at 1, 3, 5, and 7 years after septic and aseptic index revision surgery, with re-revision for any reason as endpoint. 2-stage revisions and permanent septic Girdlestones were excluded from the survival analysis, as re-revision is a non-reliable outcome measure for these groups (e.g., 2-stage revisions always come with a re-revision). A multivariable Cox regression analysis was performed, adjusted for the following confounders: age, ASA class, previous surgeries, and diagnosis (osteoarthritis/non-osteoarthritis). The proportionality hazard assumption was checked by log-minus-log curves for all confounding factors, and the assumption of proportionality was met [21]. Body mass index (BMI) could not be included as a confounder in the multivariable analysis, because it was only registered since 2014. A sensitivity analysis was performed using data from 2014–2022 only, where BMI was added as confounder.

Furthermore, a multinomial regression analysis was performed to assess the correlation between revision-to-primary ratio and re-revision. This analysis was performed to assess whether the decided cut-off point (25 rTHAs/year) had any influence on the results. Differences in mortality between groups were evaluated using a Kaplan–Meier analysis.

A difference in revision rate of 5% points or higher was deemed clinically relevant. For all tests, a 2-tailed significance level of 0.05 was used. Results were reported as hazard ratios (HR) with 95% confidence intervals (CI). The statistical software program SPSS version 24.0 (IBM Corp, Armonk, NY, USA) was used.

### Ethics, data sharing plan, funding, use of AI, and disclosures

The scientific advisory committee of the LROI granted approval for this study. In accordance with the Dutch Medical Research Involving Human Subjects Act (WMO), ethical approval was not necessary as the data used were fully anonymized. The study received no financial support, and the authors reported no conflicts of interest. Complete disclosure of interest forms according to ICMJE are available on the article page, doi: 10.2340/17453674.2025.44331

## Results

**Figure 1:**
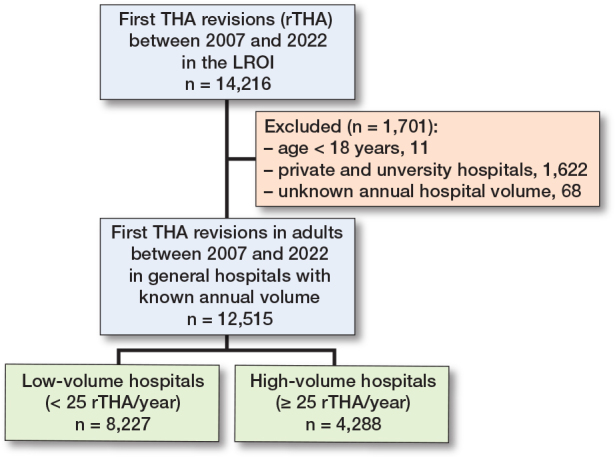
Flowchart of patient inclusion.

We included all first rTHAs performed between 2007 and 2022 (n = 14,216 rTHAs). Patients younger than 18 years were excluded (n = 11). To create more representative and generalizable results, only general hospitals were analyzed in this study, accounting for 88% of all procedures in the Netherlands. Patients treated in hospitals with unknown annual volume were also excluded (e.g., first revision in 2007, the first year of national registration). The final cohort consisted of 12,515 procedures (Figure 1), performed in 71 hospitals. The median follow-up was 4.6 years, ranging from 0 days to 14.9 years. 71 general hospitals were included with a mean of 176 procedures (range 5–1,065).

### Patient, procedure, and prosthesis characteristics

8,227 first rTHAs (66%) were performed in hospitals with a low revision volume versus 4,288 rTHAs (34%) in high-volume hospitals. Procedures in high-volume hospitals were performed in patients who were generally younger, with a higher ASA class, and had more frequently undergone previous surgery of the affected hip. High-volume hospitals more frequently opted for cemented fixation (43% vs 36%) (Table 1). 83% of revisions in low-volume hospitals were aseptic, compared with 75% in high-volume hospitals. When evaluating septic subgroups, high-volume hospitals performed 2-stage revisions more often (4.5% vs 2.3%), and DAIR procedures too (15% vs 10%).

**Table 1:** Patient and procedure characteristics of total hip arthroplasty first revisions in general hospitals in the Netherlands from 2007 to 2022. Values are count (%)

Item	Low volume (< 25/year)	High volume (≥ 25/year)	Total ^a^
Total number	8,227 (66)	4,288 (34)	12,515 (100)
Sex			
Female	5,181 (63)	2,665 (62)	7,846 (63)
Male	3,040 (37)	1,617 (38)	4,657 (37
Age (years)			
< 60	1,097 (13)	706 (17)	1,803 (14)
60–74	3,907 (48)	2,076 (48)	5,983 (48)
≥ 75	3,223 (39)	1,506 (35)	4,729 (38)
ASA classification			
I	887 (11)	405 (9.4)	1,292 (10)
II	4,577 (56)	2,506 (58)	7,083 (57)
III–IV	2,415 (29)	1,351 (32)	3,766 (30)
Body mass index			
Underweight (<18.5)	78 (0.9)	35 (0.8)	113 (0.9)
Normal weight (18.5–25)	1,871 (23)	1,251 (29)	3,122 (25)
Overweight (>25–30)	2,355 (29)	1,571 (37)	3,926 (31)
Obesity (>30–40)	1,395 (17)	1,099 (26)	2,494 (20)
Morbid obesity (> 40)	123 (1.5)	95 (2.2)	218 (1.7)
Diagnosis			
Osteoarthritis	6,735 (82)	3,528 (83)	10,263 (82)
Non-osteoarthritis	1,368 (17)	680 (16)	2,048 (16)
Previous surgeries**^a^**			
Yes	496 (6.0)	320 (7.5)	816 (6.5)
No	7,301 (89)	3,742 (87)	11,043 (88)
Smoking			
Yes	592 (7.2)	373 (8.7)	965 (7.7)
No	5,143 (63)	3,646 (85)	8,789 (70)
Fixation			
Cemented	2,767 (34)	1,739 (41)	4,506 (36)
Cementless	4,398 (54)	1,989 (46)	6,387 (51)
Hybrid			
acetabulum cemented	310 (3.8)	219 (5.1)	529 (4.2)
femur cemented	213 (2.6)	85 (2.0)	298 (2.4)
Articulation			
Ceramic-on-ceramic	351 (4.3)	50 (1.2)	401 (3.2)
Ceramic-on-metal	21 (0.3)	18 (0.4)	39 (0.3)
Ceramic-on-PE	2,545 (31)	1,149 (27)	3,694 (30)
Metal-on-ceramic	2 (0.0)	2 (0.0)	4 (0.0)
Metal-on-metal	72 (0.9)	43 (1.0)	115 (0.9)
Metal-on-PE	3,141 (38)	2,050 (48)	5,191 (42)
Zirconium-on-PE	536 (6.5)	279 (6.5)	815 (6.5)
Indication for first revision			
Septic revision	1,436 (17)	1,056 (25)	2,492 (20)
1-stage revision	351 (4.3)	176 (4.1)	527 (4.2)
2-stage revision	191 (2.3)	195 (4.5)	386 (3.1)
Permanent Girdlestone	66 (0.8)	29 (0.7)	95 (0.8)
Minor septic revision DAIR	794 (9.7)	640 (15)	1,434 (12)
Aseptic revision	6,791 (83)	3,232 (75)	10,023 (80)
Loosening	2,119 (26)	1,065 (25)	3,184 (25)
Periprosthetic fractures	1,386 (17)	619 (14)	2,005 (16)
Dislocation	2,334 (28)	1,062 (25)	3,396 (27)

a Numbers do not always add up due to missing values.

### Survival analysis: septic revisions

**Figure 2:**
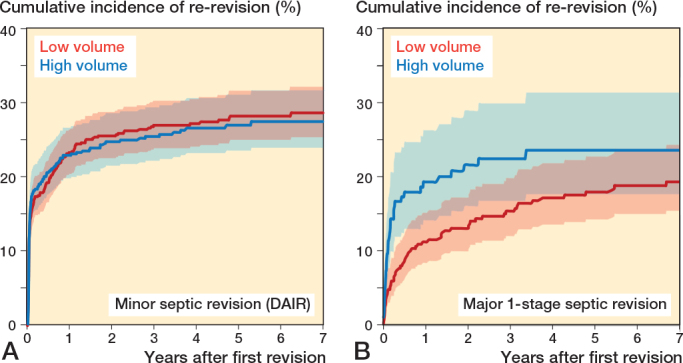
Cumulative incidence (with 95% confidence intervals) of re-revision of total hip arthroplasties in Dutch general hospitals after minor septic index revision (DAIR; A) and after major index 1-stage septic revision including Girdlestone index revisions (B).

After exclusion of 2-stage septic revisions and permanent Girdlestones, the 7-year re-revision rates for septic first revisions were 27% (CI 23.9–30.5) in high-volume hospitals and 27% (CI 24.5–30.0) in low-volume hospitals (Table 2). DAIR revisions showed 7-year re-revision rates of 28% (CI 24.0–31.6) in high-volume hospitals and 29% (CI 25.4–32.3) in low-volume hospitals (Table 2, Figure 2). For major septic 1-stage revisions (exchange of fixed components), the 7-year re-revision rates were 24% (CI 17.8–34.4) in high-volume hospitals and 19% (CI 15.5–24.4) in low-volume hospitals (Table 2, Figure 2).

**Table 2:** Cumulative incidence of re-revision after septic first revision THAs performed in 2007–2022 in Dutch general hospitals (n = 2,492)

Item	Low volume (< 25/year) % (CI)	High volume (≥ 25/year) % (CI)
Septic first revision **^a^**		
1 year	21 (18.7–23.5)	23 (19.9–25.7)
3 years	25 (22.3–27.4)	25 (22.3–28.5)
5 years	26 (23.8–29.1)	27 (23.6–30.1)
7 years	27 (24.5–30.0)	27 (23.9–30.5)
*Sub-analyses*		
Minor septic revision (DAIR)		
1 year	23 (20.5–26.4)	23 (19.9–26.6)
3 years	27 (23.9–30.3)	26 (22.3–29.3)
5 years	28 (25.0–31.7)	27 (23.6–31.0)
7 years	29 (25.4–32.2)	28 (24.0–31.6)
1-stage septic revision		
1 year	11 (8.4–15.2)	19 (14.2–26.3)
3 years	15 (12.0–19.9)	22 (16.9–30.0)
5 years	18 (14.3–22.8)	24 (17.8–31.4)
7 years	19 (15.5–24.4)	24 (17.8–34.4)

a Permanent Girdlestone and 2-stage revisions are excluded.

CI = 95% confidence interval.

In the multivariable analysis, high-volume hospitals showed a hazard ratio (HR) of 1.1 (CI 0.9–1.3) for re-revision after septic first revisions, HR 1.0 (CI 0.8–1.2) after minor septic revisions (DAIR), and HR 1.6 (CI 1.1–2.4) after major 1-stage septic revisions (Table 3). Crude analyses showed comparable results.

**Table 3:** Risk of re-revision after septic and aseptic revisions in low-volume (< 25/year) and high-volume (≥ 25/year) hospitals: multivariable Cox regression analyses. Values are adjusted hazard ratio (HR) for high-volume with low-volume as reference

Type	Adjusted hazard ratio (CI)^a^
Septic revisions**^b^**	1.1 (0.9–1.3)
Aseptic revisions	1.2 (1.0–1.3)**^c^**
Septic sub-analyses	
Septic 1-stage revisions	1.6 (1.1–2.4)**^c^**
Septic minor revisions (DAIR)	1.0 (0.8–1.2)
Aseptic sub-analyses	
Loosening	1.1 (0.9–1.4)
Dislocation	1.1 (0.9–1.4)
Periprosthetic fracture	1.1 (0.8–1.5)

a Adjusted for age, ASA score, previous surgeries, and diagnosis. Periprosthetic fracture was adjusted for age, ASA class, previous surgeries, diagnosis, and fixation.

b Septic revisions after exclusion of permanent Girdlestone and 2-stage septic revisions.

c P < 0.05.

CI = 95% confidence interval.

### Survival analysis: aseptic revisions

**Figure 3:**
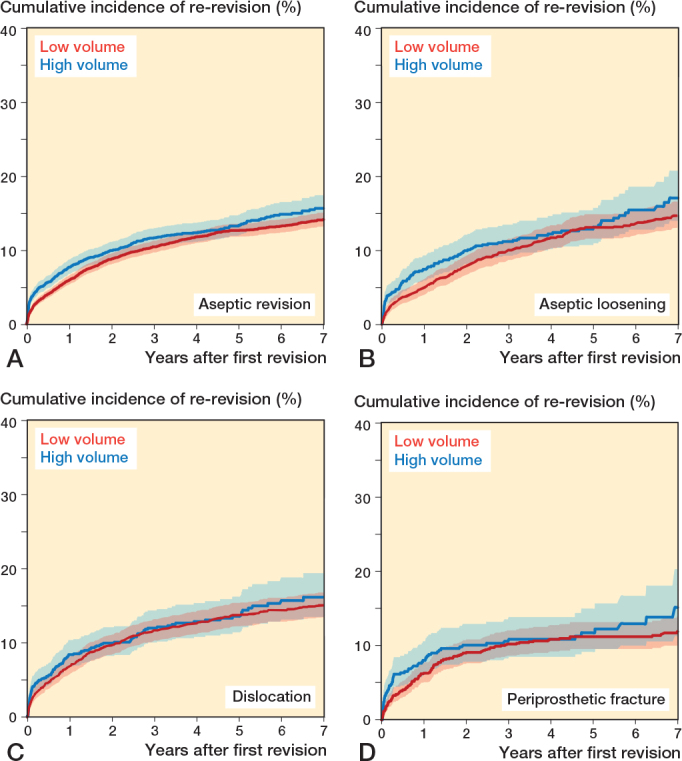
Cumulative incidence of re-revision (with 95% confidence intervals) after aseptic revision total hip arthroplasties (A), and after aseptic loosening (B), dislocation (C), and periprosthetic fracture (D) in Dutch general hospitals.

At 7 years’ follow-up, crude re-revision rates for aseptic loosening were 17% (CI 14.1–20.8) in high-volume and 15% (CI 13.3–16.7) in low-volume hospitals; for dislocation, 16% (CI 13.6–19.5) and 15% (CI 13.7–18.8); and for periprosthetic fracture, 15% (CI 11.4–20.4) and 12% (CI 10.2–14.0), respectively (Figure 3, Table 4, see Supplementary data).

**Table 5:** 7-year mortality after septic and aseptic revision THAs performed in 2007–2022 in Dutch general hospitals (n = 12,215)

Item	Low volume		High volume	
	(< 25/year)		(≥ 25/year)	
	% (CI)	No. at risk	% (CI)	No. at risk
Overall	22 (20.5–22.9)	3,067	19 (16.6–20.6)	511
First revision				
Septic	22 (19.5–25.6)	372	19 (15.4–22.8)	112
Aseptic	22 (20.4–22.8)	2,700	18 (16.2–20.6)	414

CI = 95% confidence interval.

In the multivariable analysis, HRs for high-volume hospitals were 1.1 (CI 0.9–1.4) for aseptic loosening, 1.0 (CI 0.9–1.3) for dislocation, and 1.1 (CI 0.8–1.5) for periprosthetic fracture (see Table 3).

### Mortality

Mortality rate after 7 years’ follow-up was 22% (CI 20.5–22.9) in low-volume hospitals, and 19% (CI 16.6–20.6) in high-volume hospitals. Mortality after septic first revisions was 22% (CI 19.5–25.6) and 19% (CI 15.4–22.8) in low- and high-volume hospitals. Mortality after aseptic revisions was 22% (CI 20.4–22.8) and 18% (CI 16.2–20.6), respectively (Table 5).

### Sensitivity analyses

A sensitivity analysis was performed using only data from 2014–2022 and adding BMI as confounder. This had a negligible effect on hazard ratios overall (data not shown).

An additional multinomial regression analysis, using revision-to-primary ratios, showed that an increase in the ratio resulted in slightly lower mortality rates, but did not significantly influence risk of re-revision.

## Discussion

We compared re-revision risk and mortality of septic and aseptic revisions performed in high-volume hospitals (≥ 25 rTHA/year) and low-volume hospitals (< 25 rTHA/year) in the Netherlands. High-volume hospitals showed a higher re-revision rate after septic 1-stage revisions of 24% (CI 17.8–34.4) vs 19% (CI 15.5–24.4) in low-volume hospitals. This difference was deemed clinically relevant ≥ 5%) and statistically significant. After adjustment for confounders, high-volume hospitals showed a significantly higher risk of re-revision after 1-stage septic revisions: HR 1.6 (CI 1.1–2.4). 2-stage septic revisions were more often performed in high-volume hospitals (4.5% vs 2.3%). Other indications showed no clinically relevant differences in re-revision rates: we found no evidence of a higher risk of re-revision for either volume group after minor septic revisions (DAIR), and after aseptic revisions due to loosening, dislocation, and periprosthetic fracture. There were no clinically relevant differences in mortality, for either septic or aseptic revisions.

### Previous studies

Previous studies have evaluated the association between annual rTHA volume and risk of re-revision; however, little is reported on septic rTHA specifically. Samuel et al. (2022, n = 3,057) reported lower odds of mortality in high-volume hospitals after septic rTHA (OR 0.54, CI 0.33–0.88), but re-revision risk was not reported [23]. Aseptic revisions were described more extensively: Jeschke et al. (2019, n = 16,376) reported an increased short-term risk at 1 year for low-volume hospitals (< 25 and < 53 rTHA per year) after aseptic index revisions [14]. Holleyman et al. (2024, n = 12,961) specifically analyzed first revisions due to aseptic loosening, and reported a higher risk of re-revision in low-volume hospitals [16]. Other studies evaluated revision total knee arthroplasties (rTKA): Halder et al. (2020, n = 23,644) reported a higher risk of re-revision in low-volume hospitals after aseptic rTKA [15]. In contrast, van Rensch et al. (2023) analyzed all septic and aseptic rTKAs registered in the Dutch Arthroplasty Register and concluded that rTKA early re-revision rate did not depend on hospital volume, regardless of revision type [17]. Our data on Dutch hips seems to come to the same conclusion as the latter study.

### Interpretation

Septic 1-stage first revisions showed a significantly higher risk of re-revision in high-volume hospitals, which is contrary to existing literature. Our data indicated more complex infection pathology in high-volume hospitals, which was corroborated by the higher ASA classes in high-volume hospitals. Due to local referral guidelines in the Netherlands regarding septic revisions, low-volume hospitals refer their most complex patients to high-volume hospitals for index revisions. In addition, there might be a potential difference in threshold to perform a new revision procedure in vulnerable patients in low-volume hospitals for various reasons that are not registered in the LROI, e.g., bone quality, bone loss, specific comorbid conditions. Minor septic first revisions (DAIR) showed similar re-revision risk in high- and low-volume hospitals, suggesting DAIR procedures can be safely performed by either high- or low-volume hospitals, without an increased re-revision risk for patients.

We found no clinically relevant differences in re-revision risk in high- and low-volume hospitals after aseptic rTHA. More specifically: first revisions due to aseptic loosening, dislocation, and periprosthetic fractures showed similar risk of re-revision in high- and low-volume hospitals. This suggests that these types of revisions may safely be performed with no increased risk of re-revision or mortality.

### Strengths

The strengths of this study are the completeness of the THA data in the LROI and the large real-world revision THA cohort. Our nationwide database has more than 98% completeness of THA revisions, leading to high accuracy and validity [3,24].

### Limitations

First, as mentioned earlier, reference guidelines say that the most complex patients are referred to high-volume hospitals. We have no detailed information concerning the patients referred to the high-volume hospitals. For example, high-volume hospitals are more likely to perform technically demanding procedures, such as extensive bone reconstructions in periprosthetic fractures, which inherently carry a greater risk of complications and re-revision. These patients can be more demanding due to more bone loss around the loose prosthesis, more muscle damage, or difficult to revise prostheses. Unfortunately, we were unable to obtain information on the complexity of the procedures, as this data was not recorded in the LROI.

Second, some data is missing, as BMI, smoking, and Charnley score were not registered until 2014. Therefore, we could not adjust for BMI as confounder in the main multivariable analysis. However, a sensitivity analysis on a subset of the data has been performed, including BMI as confounding variable. Additionally, the dataset does not include information regarding PROMs or conservative treatment of infected arthroplasties (e.g., suppressive antibiotics or monitored low-grade infection), and probably not all failed implants are being revised (e.g., vulnerable patients).

Third, as only (re-)revision rate is evaluated in this study, the actual number of failed implants could be underestimated. In addition, our data contained no information regarding the underlying pathogens, which could differ between volume groups (e.g., easy or difficult to treat, antibiotic resistance).

Fourth, the last factor that could obscure the results is the unknown data concerning surgeon volume. This means that the caseload or experience per surgeon is not known and therefore cannot be related directly to the annual hospital volume in this study, but could potentially influence the results. However, we believe that, within a hospital, revision surgeries are generally assigned to the surgeon with the most experience and expertise in performing these complex procedures. As a result, the lack of data on individual surgeon volume may have a limited impact on our findings, as the expertise required for revision procedures is likely concentrated among a smaller number of experienced surgeons within each hospital.

### Implications for future research

While the assessment of re-revision rates is very insightful, it is merely one aspect of a complex topic. Evaluation of other aspects in the future is recommended, e.g., the association between annual revision volume and PROMs.

Future studies could also focus on further evaluation of infection treatment. Low-volume hospitals perform fewer re-revisions due to infections, but it remains unclear whether these patients are cured or if they are treated with suppressive antibiotics or have monitored low-grade infection.

### Conclusion

There was no difference between high-volume hospitals and low-volume hospitals regarding septic and aseptic revision THA. Risk of re-revision was significantly higher in high-volume hospitals after 1-stage septic revisions. 2-stage revisions were more often needed in high-volume hospitals, most likely due to a more complex patient population and regional referral guidelines. We found no increased risk of re-revision after aseptic first revisions (e.g., loosening, dislocation, and periprosthetic fracture) in low- compared with high-volume hospitals.

*In perspective,* based on the current reference guidelines in The Netherlands, it seems that a minimum caseload may not be required in first-time revisions of THA.

### Supplementary data

Table 4 and the Directed Acyclic Graph are available as supplementary data on the article page, doi: 10.2340/17453674.2025.44331

## Supplementary Material



## Notes

Authors contributed to: (1) study design and study protocol, (2) gathering data, (3) analyzing data, (4) initial draft, and (5) final draft. MCV and RMP contributed to: 1–5; BJvD contributed to: 1–3, 5; AD contributed to: 3–5; WPZ contributed to: 1, 2, 4, 5; LNvS and BWS contributed to 1, 2, 5.

N. Veeger (statistician at Medical Centre Leeuwarden) assisted in data transformations and created the volume variables needed for our analysis.  

Handling co-editors: Keijo Mäkelä and Philippe Wagner

*Acta* thanks Samo Karl Fokter and Tina Strømdal Wik for help with peer review of this manuscript.
