# Supplementary material for: High-volume hospitals do not perform better than low-volume hospitals in septic and aseptic revision total hip arthroplasty: an analysis of re-revision risk and mortality based on the Dutch Arthroplasty Register
Source: Acta Orthop. 2025 Aug 15;96:625–31. doi: 10.2340/17453674.2025.44331 (PMC12357176; doi:10.2340/17453674.2025.44331)
Supplement: Supplementary file 1 [file ActaO-96-44331-s1.pdf]

Supplementary data

Table 4. Cumulative incidence of re-revision after aseptic first revision THAs performed in 2007–2022 in Dutch general hospitals (n = 10,023)				
	**Low volume (<25/year)**		**High volume (≥25/year)**	
	%	CI	%	CI
**Aseptic first revision**				
1 year [a]	5.9	5.4–6.5	7.7	6.8–8.7
3 years	11	9.7–11.2	12	10.5–12.9
5 years	13	11.9–13.5	13	12.1–14.8
7 years	14	13.3–15.1	16	14.1–17.4
**Sub analyses**				
**Aseptic loosening**				
1 year	5.0	4.2–6.1	7.5	6.0–9.3
3 years	10	8.9–11.5	11	9.4–13.5
5 years	13	11.7–14.8	13	11.1–15.8
7 years	15	13.3–16.7	17	14.1–20.8
**Dislocation**				
1 year	6.9	5.9–8.0	8.5	6.9–10.4
3 years	12	10.5–13.2	12	10.1–14.4
5 years	14	12.4–15.4	14	11.5–16.3
7 years	15	13.7–18.8	16	13.6–19.5
**Periprosthetic fracture**				
1 year	6.3	5.1–7.8	8.1	6.1–10.6
3 years	10	8.7–12.1	11	8.3–13.6
5 years	11	9.6–13.2	12	9.2–15.1
7 years	12	10.2–14.0	15	11.4–20.4

[a] significant

CI = 95% confidence interval

FIGURE

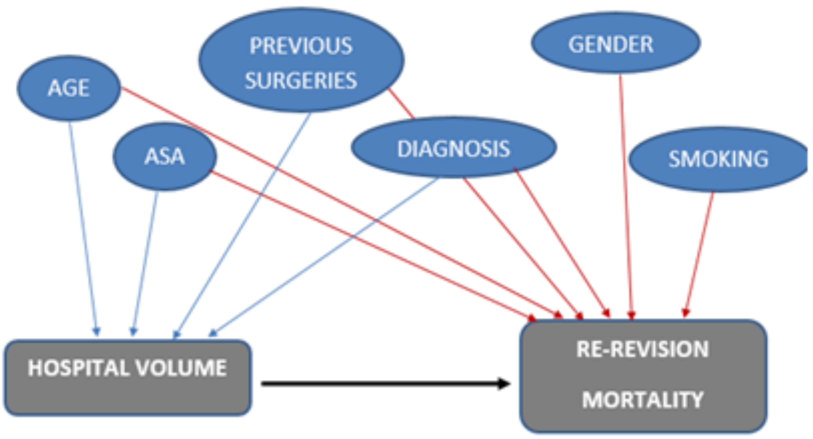

Figure 2. Directed Acyclic Graph (DAG)
